# Predictors of Dropout in Disordered Gamblers in UK Residential Treatment

**DOI:** 10.1007/s10899-019-09876-7

**Published:** 2019-07-13

**Authors:** Amanda Roberts, Raegan Murphy, John Turner, Steve Sharman

**Affiliations:** 1 School of Psychology, College of Social Science, University of Lincoln, Brayford Pool, Lincoln, Lincolnshire LN6 7TS UK; 2 School of Applied Psychology, UCC Enterprise Centre, University College Cork, North Mall, Cork, Ireland; 3 School of Psychology, University of East London, Stratford, London, E15 4LZ UK

**Keywords:** Gambling, Disordered gambling, Residential treatment, Inpatient, Treatment dropout

## Abstract

Within the cohort of individuals who seek treatment for disordered gambling, over half fail to complete treatment. The current study sought to identify predictors of treatment dropout in a sample of gamblers attending a residential treatment facility for disordered gamblers in the UK and to report differences in voluntary and enforced dropout. Data on 658 gamblers seeking residential treatment with the Gordon Moody Association (GMA) was analysed, collected between 2000 and 2015. Measurements included demographic data, self-reported gambling behavior, (including the Problem Gambling Severity Index), mental and physical health status, and a risk assessment. Binary logistic regression models were used to examine predictors of treatment termination. Results confirm a high percentage of treatment dropout among disordered gamblers (51.3%). Significant predictors of treatment dropout included older age of the client, higher levels of education, higher levels of debt, online gambling, gambling on poker, shorter duration of treatment, higher depression, experience of previous treatment programmes and medication, and adverse childhood experiences. Within non-completers, significant predictors of enforced dropout included lifetime homelessness, less debt, sports gambling, depression and lifetime smoking. Those who were on a longer treatment programme and had previously received gambling treatment or support were less likely to be asked to leave. Clinicians working in inpatient support need to be aware of the increased psychopathogical and psychosocial problems in those who are at risk of termination and make attempts to retain them in treatment and increase patient compliance.

## Introduction

There has been a considerable rise in gambling related harm since the global acceleration of gambling opportunities (Petry et al. 2017). Approximately 2.3% of the world’s population experience problems with gambling (Williams et al. 2012) and in the UK, the Health Survey for England reported problem gambling prevalence figures of 0.6–0.7% with a further 3.9% categorised as at-risk gamblers (Conolly et al. 2017). Disordered gambling can be considered concomitant with a variety of psychopathogical and psychosocial problems and harms (Cowlishaw and Kessler 2016). Of those individuals who experience disordered gambling, it has been estimated that 80–95% never obtain formal support for their problems (Volberg et al. 2006). Within the small number of individuals who do seek treatment, approximately 45–50% will drop out or prematurely terminate the programme before treatment has been completed (Melville et al. 2007; Ronzitti et al. 2017). Furthermore, when gamblers do engage with treatment, they habitually re-schedule, cancel, or fail to attend sessions (Toneatto 2005). However, evidence confirms that individuals who complete treatment find it extremely beneficial, and psychotherapeutic interventions for pathological gamblers cultivate substantial improvement in outcome and symptom relief (Pallesen et al. 2005). Consequently, it is important for treatment providers and clinicians to foster compliance and retain problem gambler clients in clinic (Melville et al. 2007).

A number of studies have identified variables associated with dropout from outpatient disordered gambling treatment programmes including neuroticism (Echeburúa et al. 2001), impulsivity (Leblond et al. 2003), alcohol consumption (Echeburúa et al. 2001), PTSD and personality disorders (Maniaci et al. 2017), onset and duration of gambling (Milton et al. 2002), debt (Brown 1986), age (Echeburúa et al. 2001) and unemployment (Hodgins et al. 2004). To our knowledge, there are very few studies that have investigated variables related to drop out in inpatient gambling treatment programmes, and none in the UK. Most gambling disorder treatment studies focus on outpatient therapy (Ledgerwood and Arfken 2017), although there are a small number of exceptions to the rule that concentrate on inpatient therapy (e.g. Blaszczynski et al. 1991; Ladouceur et al. 2006; Sander and Peters 2009). Often, inpatient addiction treatment is sought when individuals have failed outpatient treatment, or whose problems are too acute to be managed in the community and need higher levels of care (Passetti et al. 2011; Ledgerwood and Arfken 2017). A previous study with 134 inpatients revealed that such individuals had more severe gambling problems and gambling-related problems than those receiving outpatient services (Ladouceur et al. 2006).

Previous research examining reasons for treatment dropout has focussed on person-centred retrospective predictors such as the presence of cluster B personality disorders (Pelletier et al. 2008), individual coping strategies (Melville et al. 2007), or personal cognitive distortions relating to gambling (Grant et al. 2004). An Australian qualitative study identified indicators of treatment dropout encompassing a wide range of individual factors, from treatment task compliance to psychological factors (Dunn et al. 2012), however although such studies can inform treatment drop out where the individual actively chooses to leave treatment, to our knowledge no studies have differentiated between treatment drop out where the individual has made an active choice to leave, and treatment drop out where the decision to terminate treatment was made by someone other than the individual, i.e. asked to leave by the treatment support service. Therefore, reasons for enforced drop out are hithero unexplored.

The present study is the first of its kind to investigate treatment dropout among individuals attending a residential inpatient treatment programme at the Gordon Moody Association (GMA) in the UK and to examine differences between voluntary and enforced dropout. GMA provides the UK’s only gambling specific inpatient residential facility.^1^ Those in residential care are often at a more chronic and severe stage of addiction (Ledgerwood and Arfken 2017); developing a better understanding of the specific socio-demographic and clinical variables that predict treatment termination will inform gambling support services both nationally and internationally, and increase patient compliance and retention in residential care.

The primary aims of this study were to identify predictors of treatment dropout in a sample of gamblers attending a residential treatment facility for disordered gamblers, and to establish any differences between voluntary and enforced dropout.

More specifically, the study aimed to:

1. Examine the frequency of treatment dropout among individuals seeking residential treatment for gambling disorder.
2. Identify significant predictors of treatment dropout.
3. Evaluate differences in sociodemographic variables and clinical characteristics between voluntary and enforced dropout.

## Method

### Participants

Data was collected from all applicants to GMA at both GMA sites (Dudley, West Midlands and Beckenham, South-East London) on entry to the clinic between January 2000 and November 2015. Due to the residential nature of the rehabilitation programme, GMA long-term residents are male, hence only data from male gamblers with treatment outcome data are reported here (*n* = 658). Mean age at point of entry was 34.82, (*s.d.* 9.98; Min 17, Max 70).

Of those who reported their ethnicity (n = 641), 88% (n = 566) identified as being White, White Irish, or White Other. Overall, 48.8% of the sample had children, 31.4% suffered from mental ill health and 23.3% had previously received treatment for their mental ill health, with depression the most commonly reported disorder (84%). Living with family was the most commonly identified current accommodation (33.2%), followed by private rented (16.5%) and sleeping rough (11.2%). The most common highest educational attainment was above GCSE (High school equivalent) (50.4%). No educational qualifications were reported by 20.6% of the sample.

The mean number of gambling activities engaged in was 4.59 (s.d. 2.95, range 0–18, n = 637). Drinking at levels which exceed recommended safe consumption levels [> 14 units per week (112 g OH), Department of Health (2016)] was evident in 37% of the sample, 61.4% were current smokers, and 31.3% disclosed recreational or habitual use of non-prescription drugs.

### Participants and Procedure

#### Socio-demographics

Socio-demographic measures used in the regression models (categorisation in parentheses) included; age (17–25, 26–35, 36+); ethnicity (white, other), education (less than high school, high school, some post-school education or higher) and amount of outstanding debt (< £10,000, £10,000–£50,000, > £50,000).

#### Gambling and Disordered Gambling

The PGSI (Problem Gambling Severity Index) was used to measure disordered gambling. The PGSI is a widely used nine item scale for measuring the severity of gambling problems in the general population developed from a subset of items from the Canadian Problem Gambling Inventory (CPGI) (Ferris and Wynne 2001). The scale is made up of four questions which assess problematic gambling behavior and five which assess adverse consequences of gambling and is scored out of 27. The items are scored from 0 to 3 (0 = never, 1 = sometimes, 2 = most of the time, 3 = almost always). Scores show whether gambling should be considered a problem; high scores usually mean serious problems. The PGSI has adequate reliability in terms of both internal consistency (Cronbach’s α scores of 0.84) and test–retest reliability (Cronbach’s α scores of 0.78) (Ferris and Wynne 2001).

#### Treatment

Participants underwent treatment for 3, 6 or 9 months, depending on date of entry into the programme, and were also asked if they had ever been in gambling or another treatment programme before. During their time in the treatment programme^2^ residents can expect help and support to address all their problems related to gambling, including health, legal, career, accommodation and debt advice. Treatment dropout was defined as leaving treatment before the full completion of the treatment programme at GMA. Reasons for leaving the programme were coded as, chose to leave (voluntary), or were asked to leave (forced). Of those who were asked to leave, reasons were varied but included continued gambling, or bringing alcohol into the residence.

#### Service-Specific Questionnaires

As part of a larger clinical assessment, individuals completed service specific ‘audit’ measures including:

##### *Gambling Audit*

Individuals were asked to state their main types of gambling activity before entering the clinic (betting on horse racing, online gambling, gaming machines, using fixed odds betting terminals and engaging in sports betting).

##### *Need Audit*

Questions were asked about specific illness and disability, mental health, and physical health. Individuals were specifically asked if they had ever suffered from mental ill health (other than disordered gambling), alcohol or drug addiction that led to problems or being hospitalised, and if they had ever smoked. They were also asked if they had ever received treatment for a mental health disorder, medication, have or have had any physical health issues.

##### *Life Audit*

Questions were asked about significant life events such as parental divorce or separation, assault during childhood, homelessness, and if they had ever attempted suicide.

Answers to questions on the audits used in the present analysis derived into a yes/no binary. Audits were completed in a one-to-one session with a member of GMA staff. For further details of the clinical assessment and the service specific measures, see Sharman et al. (2019, supplementary material).

### Statistical Analysis

Case files were electronically redacted to ensure anonymity and coded into SPSS. A series of binary logistic regressions and one linear regression were conducted with variables shown to be associated with dropout (age, education, employment, relationship status, stressful life events (see “Appendix”: based on variables listed in Melville et al. 2007). Models assessed the statistical association between both binary outcome variables (treatment completion vs. treatment dropout; voluntary dropout vs. enforced dropout) and the independent variables identified. Comparison (reference) groups for each on the analyses are shown on the right-hand side of each table. In all cases, each variable was entered into a separate model as there was no predetermined order of variables. The variables entered into the binary logistic models are shown in the “Appendix”. We report these findings below in Tables 1, 2, and 3 but highlight only the statistically significant results.

**Table 1:** Differences in sociodemographic and gambling variables between those that completed and those that terminated treatment

Variable	Completed treatment (N = 321)		Terminated treatment (N = 337) (dropped out)			Comparison group
	% (n)	OR	% (n)	χ^2^	OR (CI)	
*Demographics*						
Age at commencement of treatment programme (age 26–35)	49.1 (113)	1	50.9 (117)	4.01 (*p* = 0.045)	1.580 (1.010–2.472)*	Age at commencement (age 17–25)
Age at commencement of treatment programme (age 36+)	50.6 (91)	1	49.5 (89)	7.59 (*p* = 0.006)	2.061 (1.232–3.446)**	Age at commencement (age 17–25)
Education	54.6 (294)	1	45.4 (244)	6.38 (*p* = 0.000)	1.776 (1.137–2.772)**	No education
Ethnicity	48.8 (312)	1	51.2 (327)	0.342 (*p* = 0.559)	0.866 (0.535–1.403)	Non-White
Debts	55.9 (291)	1	44.1 (230)	4.86 (*p* = 0.027)	1.544 (1.050–2.271)**	< £10,000 (lowest debt)
Ever homeless	48.74 (321)	1	51.21 (337)	0.1 (*p* = 0.752)	1.056 (0.755–1.477)	Never homeless
*Gambling activity*						
Online gambling	48.8 (309)	1	51.2 (324)	4.77 (*p* = 0.029)	1.443 (1.038–2.005)**	No online gambling
Horse racing	49.7 (171)	1	50.3 (173)	0.07 (*p* = 0.791)	1.042 (0.767–1.415)	No horse racing
Gaming machines	46.1 (107)	1	53.9 (125)	1.181 (*p* = 0.277)	0.837 (0.608–1.153)	No gaming machines
Poker	48.78 (321)	1	51.21 (337)	12.25 (*p* = 0.000)	3.098 (1.645–5.835)***	No poker
FOBT (fixed odds betting terminal)	50.8 (124)	1	49.2 (120)	0.643 (*p* = 0.423)	1.138 (0.829–1.562)	No FOBT
Sports	48.78 (337)	1	51.21 (321)	0.063 (*p* = 0.802)	1.047 (0.729–1.504)	No sports betting

*p ≤ 0.05; **p ≤ 0.01

**Table 2:** Differences in treatment, clinical and childhood adversity variables between those that completed and terminated treatment

Variable	Completed treatment (N = 321)		Terminated treatment (N = 337) (dropped out)			Comparison group
	% (n)	OR	% (n)	χ^2^	OR (CI)	
*Treatment*						
Length of programme	49.8 (305)	1	50.2 (308)	9.74 (*p* = 0.002)	0.587 (0.420–0.820)**	14 weeks to 6 months
Previous treatment	49.19 (304)	1	50.8 (314)	5.037 (*p* = 0.025)	0.649 (0.445–0.947)*	No previous treatment
*Clinical*						
Any mental health disorder	50.3 (100)	1	49.7 (99)	0.213 (*p* = 0.644)	1.082 (0.774–1.514)	No mental health disorder
Medication	41 (59)	1	59 (85)	5.037 (*p* = 0.025)	0.649 (0.445–0.947)*	No medication
Depression	55.6 (309)	1	44.4 (247)	5.29 (*p* = 0.021)	1.713 (1.083–2.711)*	No depression
Smoking	58.8 (181)	1	46.9 (160)	2.087 (*p* = 0.149)	0.775 (0.548–1.095)	No smoking
Drug addiction	51.4 (73)	1	48.6 (69)	0.309 (*p* = 0.578)	1.112 (0.765–1.617)	No drug addiction
Alcohol addiction	41.2 (35)	1	58.8 (50)	2.513 (*p* = 0.113)	0.687 (0.432–1.093)	Alcohol addiction
Any physical health disorder	51.1 (68)	1	48.9 (65)	0.899 (*p* = 0.343)	0.828 (0.561–1.223)	Any physical health disorder
Suicide attempt	50.7 (70)	1	49.3 (68)	0.223 (*p* = 0.637)	1.095 (0.75–1.599)	No suicide attempt
*Childhood adversity*						
Parental divorce/separation	55.5 (308)	1	44.5 (247)	4.12 (*p* = 0.042)	1.429 (1.013–2.015)*	No parental divorce/separation
Childhood adversity (violence, sexual, bullied)	48.8 (321)	1	51.1 (337)	6.81 (*p* = 0.009)	1.561 (1.117–2.182)**	No assault during childhood (violence, sexual, bullied)

	Mean	(SD)	Mean	(SD)	β	
PGSI Score (disordered gambling)	22.42 (79)	3.9	22.92 (59)	3.83	− 0.064	

*p ≤ 0.05; **p ≤ 0.01

**Table 3:** Significant differences in variables in treatment dropout: voluntary versus enforced

Variable	Individuals who chose to leave (N = 233)		Individuals who were asked to leave (N = 63)		
	% (n)	OR	% (n)	χ^2^	OR (CI)
Debts	76.9 (160)	1	23.1 (48)	6.695 (*p* = 0.01)	0.141 (0.032–0.622)**
Ever homeless	78.7 (233)	1	21.3 (63)	4.021 (*p* = 0.045)	1.815 (1.014–3.249)*
Sports gambling	78.7 (233)	1	21.3 (63)	5.483 (*p* = 0.019)	2.089 (1.128–3.87)*
Length of programme	77.7 (212)	1	22.3 (61)	5.071 (*p* = 0.024)	0.504 (0.278–0.915)*
Previous treatment	79.3 (219)	1	20.7 (57)	5.009 (*p* = 0.025)	0.416 (0.193–0.897)*
Depression	76.9 (173)	1	23.1 (52)	4.255 (*p* = 0.039)	2.87 (1.053–7.588)*
Smoking	76.9 (173)	1	23.1 (52)	3.74 (*p* = 0.053)	1.99 (0.991–3.998)*

*p ≤ 0.05; **p ≤ 0.01

## Results

### Treatment Dropout: Main Findings

Results indicate that 51.2% (n = 337) of the sample dropped out of treatment. Out of the sample who dropped out, 69.0% chose to leave, 18.7% were asked to leave, 8.0% failed the assessment (i.e. did not pass the consistent and strict criteria that has been put into place at GMA in order to ensure the safety and suitability of people attending the GMA programs and premises^3^) 2.1% were arrested or sent to prison, 0.9% were referred on to other specialised services, and 1.3% dropped out for reasons that were missing or unclear. We did not include the latter four groups in our analyses due to small numbers. The remaining 48.8% (n = 321) completed treatment.

### Treatment Dropout: Sociodemographic Variables and Gambling Modes

According to the Wald criterion [the association between the independent variables (predictors) and the criterion variable (dependent) variable (treatment drop out)], gamblers were more likely to drop out of treatment if they were older gamblers (age 26+) (vs. gamblers under the age of 26), had levels of higher education (compared to those with lower education) had higher debts (compared to those with lower debts) or if they gambled online or played poker.

### Treatment Dropout: Clinical Presentation, Childhood Adversity and Treatment

According to the Wald criterion, Gamblers who undertook the longer gambling programme (9 months) were less likely to drop out of treatment when compared to gamblers who undertook a shorter treatment programme (3–6 months). Likewise, gamblers who had received previous treatment and were taking medication were more likely to complete treatment compared to those who had/were not. Gamblers who were depressed were more likely to dropout than those not reporting depression. Additionally, gamblers who experienced parental divorce/separation and childhood adversity (experiencing violence, sexual abuse or being bullied) were more likely to drop out of treatment when compared to gamblers who did not experience such trauma. There was, however, no difference between those who completed treatment and those who dropped out of treatment regarding their PGSI (gambling severity) scores. The standardised beta regression coefficient was very small and non-significant.

### Treatment Dropout: Voluntary Versus Enforced Dropout—Reasons for Leaving

Of our sample of non-completers, 78.7% (n = 233) chose to leave. According to the Wald criterion, gamblers who were more indebted were less likely to experience enforced dropout (rather, they dropped out voluntarily). Gamblers who ever experienced lifetime homelessness were more likely to have experienced enforced dropout, as were gamblers who gambled on sports events, gamblers who smoked and gamblers who experienced depression. Gamblers who undertook the nine-month gambling programme were less likely to experience forced dropout when compared to gamblers who undertook a shorter treatment programme. Gamblers who received any previous treatment were less likely to experience forced dropout.

## Discussion

One of the primary aims of this study was to establish sociodemographic, clinical and lifetime experience differences in those who completed a residential treatment course, and those that did not. Of the sample, 51.3% (n = 337) dropped of treatment, broadly in line with previous research in outpatient settings (Melville et al. 2007; Ronzitti et al. 2017). Significant predictors of treatment dropout included age of the client (over 26), higher education, higher debts, online gambling, gambling on poker, shorter duration of treatment, depression, experience of previous treatment programmes and medication, and adverse childhood experiences.

Disordered gambling has been shown to be coexistent with a range of psychopathogical and psychosocial problems and harms (Cowlishaw and Kessler 2016). Our findings suggest that multi-morbidities and more acute psychopathology may have significant implications for future treatment attrition. Individuals were more likely to terminate the programme if they were suffering from depression and had experienced parental divorce/separation and childhood assault. It has been proposed that the tiredness, lack of interest, and feelings of despondency suffered through depression may make individuals more likely to drop out (Melville et al. 2007). Such individuals may need more time and effort in treatment due to additional care for their comorbid depression. Likewise, several studies have shown a high incidence of childhood maltreatment, trauma and abuse in disordered gambling groups (Roberts et al. 2017); these early experiences may influence an individual’s attachment style, principally the capacity to formulate and sustain a trusting relationship with other individuals, and in this case, the therapist (Mahon et al. 2001). Given the necessarily intensive nature of residential treatment, failure to create a degree of trust between therapist and client would represent a significant obstacle to successful completion of a long-term treatment programme.

Conversely, gamblers who had received previous treatment for gambling and were taking mental health medication were more likely to stay in the programme. Individuals may be more likely to continue treatment if they have been previously ‘primed’ for treatment by earlier experiences or if they are accustomed to the nature of psychological treatment (see Stark 1992). Moreover, for many, treatment at GMA is seen as the ‘last chance’ when other treatment has failed. Such individuals may have a better understanding of the need to fully commit to the residential treatment programme and could offer peer support to individuals who are at risk of termination. Peer support has been shown to be beneficial to those in substance abuse treatment, especially in difficult-to-engage populations (see Tracy and Wallace 2016).

Following internal organisational reviews of programme length at GMA, the residential treatment programme was shortened initially from 9 to 6 months in 2009 and a year later in 2010 to 3 months. Participants were more likely to drop out of the programme with a shorter treatment duration. There is currently no comparable data for length of stay and drop out in residential gambling treatment, although previous studies in substance misuse treatment have shown the opposite findings; the number of patients who leave treatment is elevated in long-term compared to short-term inpatient programs (Andersson et al. 2018). However, the Transtheoretical Model (stages of change) may in part expound the findings (Prochaska et al. 1992). The model proposes that individuals progress through a series of stages that shape their motivations to change (i.e. no desire (precontemplation), through to preparing for (contemplation and preparation), acting, and maintaining changed behavior. The stage of change has been shown to influence dropout; those at the precontemplation stage are more likely to drop out (Callaghan et al. 2005). Individuals experiencing the shorter treatment programme are more likely to still in be in the precontemplation phase towards the end of the programme, whereas those in the longer treatment programme have more time to move through to the contemplation and preparation phases. Moreover, the longer treatment may allow individuals time to maintain long term goals. It may be useful for clinicians to measure readiness for change stages throughout treatment to maintain compliance. Treatment could be tailored to the measured stage. However, in-treatment factors such as available personnel resources, therapeutic competence, and patient involvement may also be related to treatment outcome, and warrant further investigation.

The study also sought to establish differences in profile of those who voluntary left treatment, and those who were asked to leave. Most individuals who left the programme chose to leave of their own accord, and only 21.3% or individuals who left experienced enforced dropout. Significant predictors of enforced dropout included individuals with less debt, no experience of previous gambling treatment or support, a preference for sports gambling and those on the shorter treatment programme. Additionally, those who were asked to leave reported more negative psychopathology than those who voluntarily left in the guise of lifetime homelessness, depression, and smoking. It is possible that engaging in behavior that resulted in being asked to leave, such as threatening staff, fighting with other residents, bringing alcohol into a dry house, and continued gambling are indicative of underlying personality disorder or psychopathology of which disordered gambling is merely symptomatic, consistent with the antisocial-impulsivist (AI) pathway identified in the Pathways Model (Blaszczynski and Nower 2002). It has been suggested that AI gamblers respond less positively to treatment (Ledgerwood and Petry 2010), therefore consideration of the individual’s overall psychopathology is significant in the prevention of treatment drop out. Drop out may indicate the need for more specialist provision for more challenging clients, less suited to broader- based residential care. Such care could mitigate against treatment failure for some subgroups and enhance overall rates of successful outcomes. For example, clinicians could apply supplementary treatment strategies such as Motivational Interviewing (a nonconfrontational psychotherapeutic approach that endeavours to shift a state of indecision or uncertainty towards a state of motivation to enable a client to make positive decisions and realise established goals in collaboration with the therapist) (Miller and Rollnick 2002) in the early phases of treatment designed to target gamblers at risk of dropout. Research has shown that Motivational Interviewing works well with gamblers (Yakovenko et al. 2015). However, it must be noted that the treatment environment may increase/decrease the risk factors for enforced drop out. For example, outpatient treatment facilities may terminate treatment for a certain number of failed appointments; which may be less likely to happen in residential care. Conversely, residential treatment programmes may enforce drop out for alcohol use, but this is less likely to be policed in outpatient clinics.

The present study is not without limitations. Measures used were assessed by self-report and did not include corroborative data. Self-report data, especially of a personal nature, carries with it distortions related to memory and retrospective reporting and demand characteristics (Rosenman et al. 2011). Also, although the data spans a large time period (analyses), it does not give us any information regarding those who did not seek treatment, therefore perhaps neglecting a subset of disordered gamblers; nor does it give us relapse rates. Risk factors that may precipitate treatment termination may not be the same as those that predict gambling relapse. Moreover, the analyses only include disordered gamblers who represent those gamblers at the more severe end of the gambling harm spectrum, hence it is unknown if our results can be generalised to gamblers in outpatient care, at-risk gamblers, or to non-treatment seeking gamblers. Furthermore, the sample in the current study was male; it is unknown if predictors of treatment dropout identified in the current study would also be generalisable to female gamblers. Further work is needed to identify predictors of treatment dropout in female gamblers.

## Conclusion

Notwithstanding these limitations however, the present study is the first to investigate dropout rates among a large group of individuals attending a residential inpatient treatment programme in the UK and to report differences in voluntary and enforced dropout. The study aids identification of individuals at risk of attrition and the basis for developing interventions to reduce this. Identification of factors that could predict treatment dropout is highly beneficial to those seeking to understand, support and provide treatment for gambling disorder, therefore increasing efficacy of treatment for those most in need of support for gambling related problems.

## Appendix

Analysis consisted of sociodemographic details including age at commencement of treatment programme, level of highest education attained, ethnicity, amount of outstanding debt, and if the individual had experienced homelessness. Gambling related variables included online gambling activity, betting on horse racing, using gaming machines, engaging in poker, using fixed odds betting terminals and engaging in sports betting. Clinical variables included length of treatment programme, whether previous treatment had been undertaken, being diagnosed with any mental health disorder, taking medication, being depressed, being a smoker, experiencing disordered gambling, experiencing drug addiction, experiencing alcohol addiction, suffering from a physical health disorder, attempting suicide, experiencing parental divorce/separation, and suffering any childhood adversity such as experiencing violence, sexual abuse and being bullied.

## Footnotes

1 In the UK, there is a restricted choice of psychological treatment available to those seeking support for problematic and disordered gambling. GamCare, the largest non-statutory treatment provider provides online and telephone-based support services, as well as one to one counselling through a network of counsellors. The NHS supported National Problem Gambling Clinic provides Cognitive Behavioral Therapy and psychodynamic psychotherapy, as well as couples therapy and psychiatric reviews. Gamblers are also able to access Gamblers Anonymous, a self-help group based on the 12-step model of Alcoholics Anonymous. The residential treatment programme is the only funded programme in the UK run exclusively for pathological gamblers.

2 In line with many other addiction treatment approaches, the GMA treatment programme is based on a cognitive behavioral approach and uses Cognitive Behavior Therapy (CBT) that helps the individual to understand how thoughts, emotions and behavior are all connected. Furthermore, during their time in the treatment programme residents can expect help and support to address all their problems related to gambling, including health, legal, career, accommodation and debt advice. Treatment included both individual and group sessions.

3 Male; 18 years and above; previous attempts to address gambling; high level of motivation; ability to live in a community and share facilities; able to live independently (cook, clean, wash etc.); ability to undertake and engage in therapeutic interventions (groups, counselling); willing to attend the full duration of the programme with no home return, and to consider further stay at the follow-on Recovery House where suitable; no specific care needs that will impact ability to complete programme; conditions re offending history/certain offence types; must be able to pay weekly rent and personal charges (× 14 weeks) of approx. £215 and £10.50 respectively via benefits or self-funds.
